# Effectiveness of anticoagulant therapy in the treatment of post-TAVI bioprosthetic thrombosis

**DOI:** 10.1186/s13019-015-0254-5

**Published:** 2015-04-01

**Authors:** Jérémy Descoux, Pierre Gautier-Pignonblanc, Andrea Innorta, Nicolas Durel, Lionel Camilleri, Pascal Motreff, Jean-René Lusson, Geraud Souteyrand

**Affiliations:** Centre Hospitalier Universitaire de Clermont Ferrand and the Faculty of Medicine of Clermont-Ferrand, Université d’Auvergne, Clermont-Ferrand, France

**Keywords:** Thrombosis, Bioprosthesis, TAVI, Anticoagulation, Echocardiography, Aortic stenosis, Male, > 80 years

## Abstract

Bioprosthetic thromboses are rarely reported in post-transcatheter aortic valve implantation (TAVI). We describe herein the case of bioprosthetic valve thrombosis in an 82-year-old patient which resolved completely after anticoagulant therapy.

## Background

Aortic valve replacement enables to reduce symptoms and improve morbidity and mortality in patients with severe aortic stenosis [1]. Transcatheter valve replacement represents a safe and well-recognized alternative in inoperable and/or high-operative-risk patients (EuroSCORE, STS) [2-4].

Antithrombotic therapy in the setting of TAVI has been empirically determined, with the most commonly recommended treatment consisting of unfractionated heparin during the procedure followed by dual antiplatelet therapy with aspirin (indefinitely) and clopidogrel (1 to 6 months) [5-7].

To date, there are no referenced cases of described valvular thrombosis in the large prospective cohorts addressing post-TAVI follow-up [8-10]. Other published reports include one case of an Edwards SAPIEN 23 mm valve dysfunction requiring re-operation [11] , one case of early transcatheter aortic valve thrombosis (2 weeks) requiring a second intervention [12]., one case of valve thrombosis which could not be treated by warfarin and requiring surgical intervention [13], one case of prosthesis thrombosis with restricted cusp movement and conserved pressure gradient with complete disappearance 10 weeks after reintroduction of anticoagulant therapy [14], one case of thrombotic aortic stenosis of an Edwards SAPIEN 26 mm valve occurring 4 months after the procedure with total recovery upon vitamin K antagonist (VKA) therapy [15].

Leetma et al. furthermore described 2 cases of refractory heart failure leading to death at respectively day 106 and day 137 following transapical TAVI [16].

Cota et al. also described 3 cases of bioprosthetic dysfunction resulting from valve thrombosis, at respectively 10, 4 and 2 months, all of which were treated with VKA therapy with *ad integrum* restitution within 2–3 months [17].

Finally, Latib et al. reported 3 cases of SAPIEN XT valve thrombosis assessed during TAVI-follow up, at respectively 6, 15 and 24 months. All three cases were treated with VKA therapy with complete restitution within 1–2 months [18].

## Case presentation

We report herein the case of an 82-year-old patient with a respective history of idiopathic thrombocytopenic purpura (ITP), type 2 diabetes, treated dyslipidemia and chronic ischemic heart disease (CD Angioplasty, AIV), recent disabling stroke, Parkinson’s disease, angina pectoris and dyspnea on exertion (NYHA 3). The patient was diagnosed with severe aortic stenosis: annulus = 23 mm, area = 0.85 cm^2^, minimal aortic insufficiency (AI), transvalvular mean gradient (MG) = 30 mmHg, with consistent LVEF at 55% in transthoracic echocardiography (TTE), pulmonary arterial hypertension (PAH) at 50 mmHg and coronary lesions for which the patient received a stent in the middle segment of the right coronary artery prior to the procedure. EUROSCORE was 26% while STS score was 2.39% risk of mortality and 14.73 % risk of morbidity or mortality.

The patient was implanted with a 26 mm Edwards-Sapien XT Valve via left femoral access without complications.

Echocardiographic control at post-procedure and at one month revealed an aortic valve area of 1.9 cm^2^, MG = 9 mmHg, with the patient showing clinical improvement.

The patient was re-admitted 5 months after the procedure for syncope and recurrent angina; upon TTE examination: LVEF = 55%, valve area = 0.8 cm^2^, MG = 56 mmHg.

The non-displaced bioprosthesis presented cusps with reduced mobility in the apical portion, with a predominant obstruction distally to the valve with visible thrombus. Of note, the patient had been under dual anti-platelet therapy (aspirin + clopidogrel) since his discharge.

At this point, the diagnosis of prosthetic valvular thrombosis was evoked.

Upon medico-surgical consensus, it was decided to introduce heparin therapy relayed by VKA treatment (oral anticoagulants).

**Figure 1:**
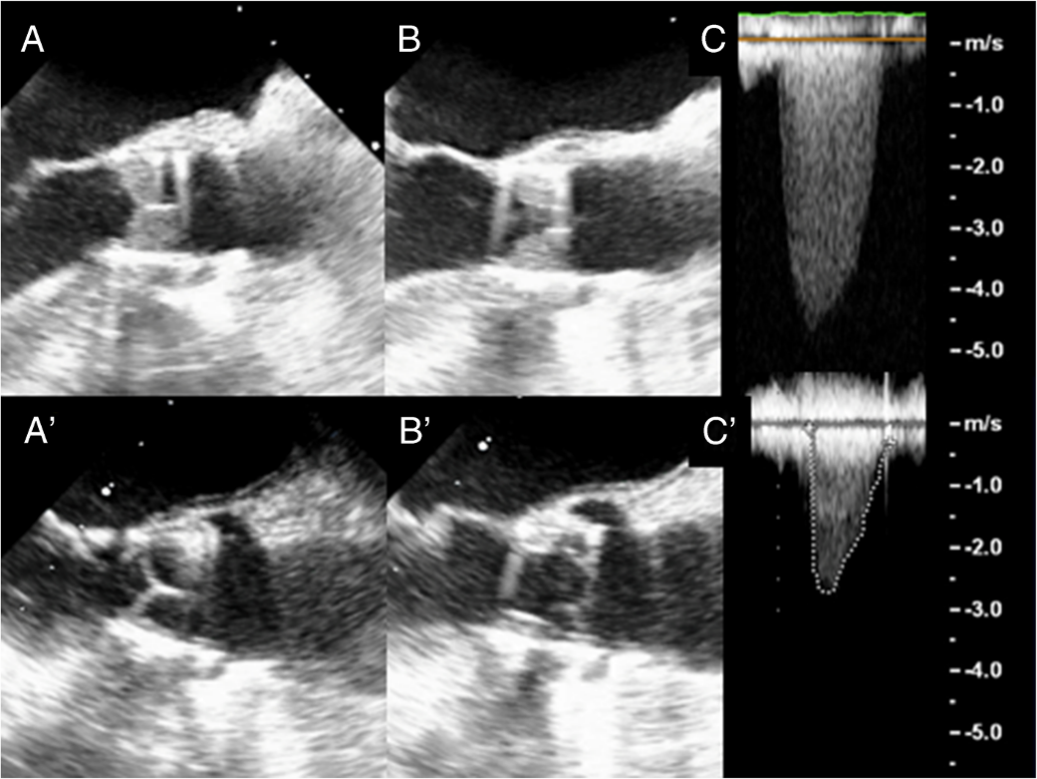
**Transesophageal echocardiogram (TEE) of the aortic bioprosthesis in diastole (A) and in systole (B), with transvalvular continuous-wave Doppler (C) revealing a mean gradient (MG) of 64 mmHg and a Vmax of 5 m/s.** Same TEE sections and continuous-wave Doppler (A’, B’ and C’) after 1 month of anticoagulant therapy: MG = 15 mmHg and Vmax = 2.7 m/s with unchanged heart rate and ejection fraction.

Upon treatment, the patient quickly improved and at one month after introduction of VKA, TTE showed LVEF = 55%, valve area = 1.8 cm^2^, Vmax = 1.73 m/s, MG = 15 mmHg, along with disappearance of the thrombosis (Figure 1 A’B’C’)

Three and a half months after introduction of anticoagulant therapy, the patient was controlled with TTE displaying a LVEF = 55%, MG = 10 mmHg with thin cusps showing no thrombosis or obstruction.

For etiological purposes, respective investigation of circulating anticoagulants, anti-B2GP1, anticardiolipids as well as a full thrombophilia work-up were performed and proved negative. A review of clopidogrel resistance by conventional methods was also performed and found to be negative. The only significant anomaly was a thrombocytopenia at 56 G/L in a context of chronic ITP.

Since then, the patient is being treated with long-term aspirin and VKA anticoagulation. Evolution remains uneventful.

## Discussion

We report herein the case of an aortic valve thrombosis after primary percutaneous intervention which was able to be treated through implementation of an anticoagulant (VKA) therapy.

To date, the durability of available bioprosthetic valve devices remains unknown.

Valve thrombosis is a distinct entity defined by the joint recommendations of the AATS/STS/EACTS in order to classify causes of postoperative morbidity and mortality [19].

The incidence of thrombosis of surgically implanted bioprostheses remains low, ranging between 0 and 1% according to the various studies [20-22].

Under the setting of TAVI, one case report described a valve restenosis at 8 months post implantation with large thrombi on 2 of the leaflets without histological evidence of endocarditis, and requiring reoperation while one other case described a 74-year-old patient under corticoid therapy who presented with early valve thrombosis 2 weeks after surgery, and requiring emergency reoperation [12]. In the first case, poor compliance to antiplatelet therapy was described.

In the 2 cases reported by Leetma et al., the diagnosis of stent-valve-thrombosis was assessed by transesophageal echocardiography and multislice computed tomography. This diagnosis was confirmed postmortem by autopsy and histology. Thrombosis occurred shortly after reduction from dual antiplatelet therapy to mono antiplatelet therapy [16].

Cota et al. also described three cases of post-TAVI restenosis under mono antiplatelet therapy at respectively 10, 4 and 2 months follow-up, for which vitamin K antagonist therapy was instituted with restitution of valvular function within 2 to 3 months [17].

In the 3 cases reported by Latib et al. of SAPIEN XT aortic restenosis assessed during TAVI follow-up at respectively 6, 15 and 24 months with recovery after 2 months of VKA treatment, no trigger or changes had been observed to explain this evolution. No thrombus was visible on successive TTE or TEE in these patients [18].

In a review by Mylotte et al., 15 cases of transcatheter heart valve thrombosis occurring 9 ± 7 months after implantation were assessed and found to be successfully treated by prolonged anticoagulation in 75% of the cases [23].

In our patient, in light of the echocardiographic aspect of valve thrombosis, it was decided to initiate anticoagulant therapy which proved to be rapidly effective. This case raises the question of anticoagulant therapy in the aftermath of a TAVI procedure. Such therapy is not recommended outside of specific indications (atrial fibrillation, etc.), with only antiplatelet therapy being recommended.

We also investigated for factors contributing to this valve thrombosis, but were unable to find a causative etiological factor. It would be interesting in the future to collate all cases of early thrombosis of percutaneous valves in order to search for elements possibly favoring such thrombosis.

## Conclusion

We report the case of early thrombosis of a percutaneous valve prosthesis effectively treated by oral anticoagulation with excellent outcome.

## Consent

Written informed consent was obtained from the patient for publication of this Case report and any accompanying images. A copy of the written consent is available for review by the Editor-in-Chief of this journal.
